# Trends in Cardiometabolic and Cancer Multimorbidity Prevalence and Its Risk With All-Cause and Cause-Specific Mortality in U.S. Adults: Prospective Cohort Study

**DOI:** 10.3389/fcvm.2021.731240

**Published:** 2021-12-09

**Authors:** Liu Yang, Jiahong Sun, Min Zhao, Costan G. Magnussen, Bo Xi

**Affiliations:** ^1^Department of Epidemiology/Shandong Provincial Clinical Research Center for Emergency and Critical Care Medicine, School of Public Health/Qilu Hospital, Cheeloo College of Medicine, Shandong University, Jinan, China; ^2^Department of Toxicology and Nutrition, School of Public Health, Cheeloo College of Medicine, Shandong University, Jinan, China; ^3^Menzies Institute for Medical Research, University of Tasmania, Hobart, TAS, Australia; ^4^Research Centre of Applied and Preventive Cardiovascular Medicine, University of Turku, Turku, Finland; ^5^Centre for Population Health Research, University of Turku and Turku University Hospital, Turku, Finland

**Keywords:** multimorbidity, mortality, cardiovascular disease, cancer, trend

## Abstract

Several prospective cohort studies have assessed the association between multimorbidity and all-cause mortality, but the findings have been inconsistent. In addition, limited studies have assessed the association between multimorbidity and cause-specific mortality. In this study, we used the population based cohort study of National Health Interview Survey (1997–2014) with linkage to the National Death Index records to 31 December 2015 to examine the trends in prevalence of multimorbidity from 1997 to 2014, and its association with the risk of all-cause and cause-specific mortality in the U.S. population. A total of 372,566 adults aged 30–84 years were included in this study. From 1997 to 2014, the age-standardized prevalence of specific chronic condition and multimorbidity increased significantly (*P* < 0.0001). During a median follow-up of 9.0 years, 50,309 of 372,566 participants died from all causes, of which 11,132 (22.1%) died from CVD and 13,170 (26.2%) died from cancer. Compared with participants without the above-mentioned chronic conditions, those with 1, 2, 3, and ≥4 of chronic conditions had 1.41 (1.37–1.45), 1.94 (1.88–2.00), 2.64 (2.54–2.75), and 3.68 (3.46–3.91) higher risk of all-cause mortality after adjustment for important covariates. Similarly, a higher risk of CVD-specific and cancer-specific mortality was observed as the number of chronic conditions increased, with the observed risk stronger for CVD-mortality compared with cancer-specific mortality. Given the prevalence of multimorbidity tended to increase from 1997 to 2014, our data suggest effective prevention and intervention programs are necessary to limit the increased mortality risk associated with multimorbidity.

## Introduction

As the global population ages, multimorbidity (e.g., two or more chronic conditions of hypertension, heart disease, stroke, diabetes, or cancer) are becoming more prevalent (1–3). Multimorbidity, which was associated with poor quality of life and reduced life expectancy (4–6), could challenge existing health care systems with unknown drug interaction and treatment burden (7).

There has been recent attention on the trends in prevalence of multimorbidity that has occurred over time. Data from the National Health Interview Survey (NHIS) found that the prevalence of multimorbidity increased from 21.8 to 26.0% among U.S. adults aged 18 years or older from 2001 to 2010 (2). Moreover, data from general practice (2004–2011) and health surveys (2001–2011) of the Netherlands have also shown a significant increased trend in the prevalence of multimorbidity among adults aged 25 years or older (from 12.7 to 16.2%, and 14.3 to 17.5%, respectively) (3). As those with multimorbidity contribute to significantly higher health care utilization and costs (8), these findings, suggesting an increasing trend in the prevalence in middle-aged and older people, underscore the urgency for effective prevention and control of multimorbidity in the general population.

Although research has shown that the existence of any of these chronic conditions can increase the risk of mortality (9–11), evidence is limited on the risk of mortality for those who have multimorbidity. Where data do exist, the findings have been inconsistent (4, 6, 12, 13). For example, while several studies have shown multimorbidity to be associated with increased risk of all-cause mortality (4, 6, 12), others have shown no association (13, 14). Moreover, limited studies have examined its association with cause-specific mortality (12).

Therefore, using data representative of U.S. adults from the NHIS (1997–2014), we aimed to examine trends in prevalence of multimorbidity from 1997 to 2014 and its association with all-cause, CVD-specific, and cancer-specific mortality until December 31, 2015.

## Methods

### Study Design and Participants

Since 1957, the NHIS has been administered by the National Center for Health Statistics to collect information on the health and lifestyle behaviors of the civilian, non-institutionalized, U.S. population using personal household interviews. Following an area probability method, a stratified and multistage sampling design was used to select a representative sample of U.S. population annually. Due to a major revision of the questionnaire in 1997, the NHIS data in this study were restricted to the participants recruited from 1997. More detailed information on the NHIS is available online (https://www.cdc.gov/nchs/nhis/index.htm). The de-identified and public NHIS data without including any protected health information are exempt from the ethical board review of Shandong University.

**Figure 1:**
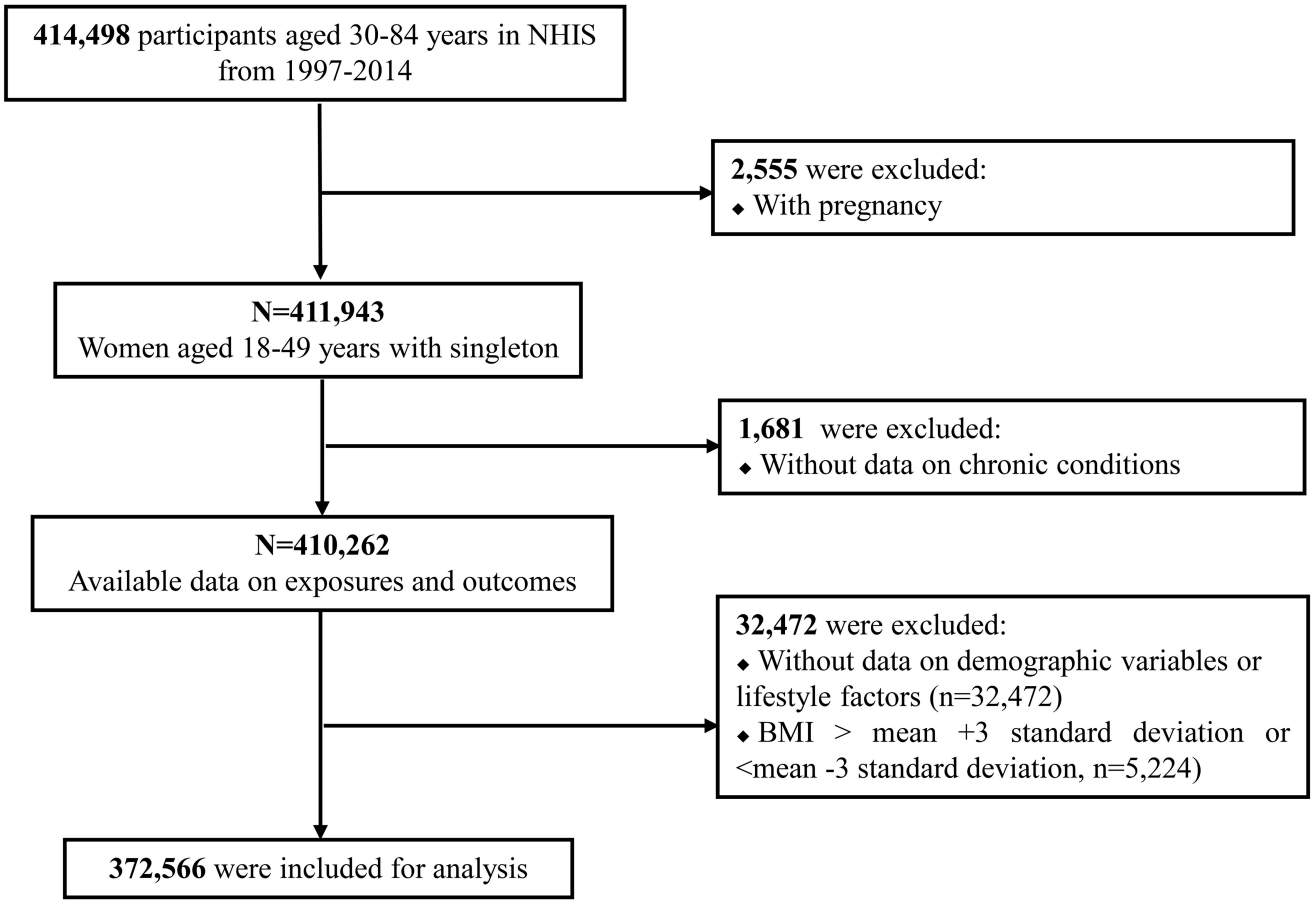
Flowchart of inclusion/exclusion of the study participants.

A total of 414,498 participants aged 30–84 years were initially identified from 18 cross-sectional household interview surveys (1997–2014). Among them, 36,708 were excluded because of pregnant women (*n* = 2,555), missing data on chronic conditions (*n* = 1,681), missing data on potentially important covariates (i.e., demographic variables and lifestyle factors, *n* = 32,472), and outliers of BMI (> mean + 3 standard deviation or < mean −3 standard deviation, *n* = 5,224), resulting in a final analytic sample of 372,566 participants. The included annual sample size ranged from 24,868 in 1997 to 25,704 in 2014. A flowchart of inclusion/exclusion of study participants is presented in Figure 1.

### Study Exposure: Chronic Conditions

The information on the chronic conditions in NHIS questionnaires was self-reported by study participants: “Have you ever been told by a doctor or other health professional that you had hypertension/heart disease (including coronary heart disease, angina, myocardial infarction, or other heart disease)/stroke/diabetes/cancer?” The number of these chronic conditions was categorized into 5 subgroups: 0 (reference group), 1, 2, 3, and ≥4.

### Study Outcome: Mortality

In this study, all-cause, CVD-specific, and cancer-specific mortality was determined by mortality records in the National Death Index to December 31, 2015. The accuracy of information on all-cause and cause-specific deaths has been validated previously (15). The *International Classification of Disease-10th Revision* codes were used to identify clinical outcomes including all-cause mortality, CVD-specific mortality (codes I00-I09, I11, I13, and I20-I51, I60-I69) and cancer-specific mortality (codes C00-C97).

### Study Covariates

Several demographic and lifestyle variables were included as potentially important covariates in this study. Demographic variables included sex, age, race/ethnicity (non-Hispanic white, non-Hispanic black, Hispanic, and others), education (<high school, high school, and >high school), and marital status (married, divorced/separated/widowed, and never married). Lifestyle variables included body mass index (BMI [kg/m^2^], calculated as self-reported weight divided by the square of height), smoking status, alcohol intake, and physical activity (PA). BMI status was categorized as underweight (<18.5 kg/m^2^), normal weight (18.5− <25 kg/m^2^), overweight (25− <30 kg/m^2^), and obesity (≥30 kg/m^2^). Smoking status was identified as never, former, and current according to self-reported answers to the following two questions: (1) Have you smoked at least 100 cigarettes in your ENTIRE LIFE? and (2) Do you NOW still smoke cigarettes? Alcohol intake was divided into lifetime abstainer, former drinker, current light-to-moderate, and heavy drinker, according to the self-reported frequency (days per week, month, or year) and quantity (drinks per day) of alcohol intake. The frequency (times per week) and duration (minutes/time) of leisure time PA (vigorous and light- to-moderate intensity) for at least 10 min at a time was self-reported. Based on the recommendation of the 2018 Physical Activity Guidelines for Americans that at least vigorous PA for 75 min/week or moderate PA for 150 min/week or equivalent combination of both, participants were categorized as meeting the recommended PA guidelines or as being physical inactive (16).

### Statistical Analyses

Baseline characteristics of categorical variables across the 5 categories of chronic conditions (0 [reference group], 1, 2, 3, and ≥4) were presented as percentages and differences between groups were examined using χ^2^ analysis Continuous variables including age and BMI were presented as mean ± standard deviation, minimum, and maximum, and the differences between groups were examined using variance analysis. We calculated the crude and age-standardized prevalence of specific chronic condition and multimorbidity to examine the trends of these prevalence estimates over time from 1997 to 2014. The age-standardized prevalence was estimated using the age distribution of the U.S. population in 2010 (17). In total, 47% of adults were aged 30–49 years, 40% were aged 50–69 years, and 13% were aged 70–84 years, and correspondingly, weights of 0.47, 0.40, and 0.13 were used to calculate age-standardization prevalence estimates in each survey (1997–2014). Multivariable logistic regression models were used to estimate the corresponding *P* values to examine the trends in the prevalence of specific chronic condition and multimorbidity over time, with adjustment for sex, age, race/ethnicity, education, marital status, BMI, smoking status, alcohol intake, and PA. Subgroup analyses were performed by age, sex, and ethnicity/race. Given that subgroup analyses created a multiple testing problem, Bonferroni method was used to correct *P* values and *P* < 0.0006 (0.05/81) was considered statistically significant.

After the test of proportional hazards assumption (meeting the assumption) using Kaplan-Meier survival curves for each outcome, Cox proportional hazards regression models were used to estimate the hazard ratios (HRs) with 95% confidence intervals (CIs) of the association between multimorbidity and all-cause, CVD-specific, and cancer-specific mortality among U.S. adults with no chronic condition as the reference. Considering the effect of potential confounding variables on the association, three models were conducted consecutively, including: Model 1: adjusted for sex, age, and race/ethnicity; Model 2: variables in Model 1 plus education and marital status; and Model 3: variables in Model 2 plus BMI, smoking status, alcohol intake, and PA. Based on the index of AIC values, we compared the three models for each outcome and selected Model 3 as the best suited model. Subgroup analyses were conducted stratified by age, sex, race/ethnicity, education, marital status, weight status, smoking, alcohol intake, and PA. In addition, we performed the analysis by excluding participants with cancer at baseline to assess the association between cardiometabolic morbidities and mortality. To test the robustness of results, two sensitivity analyses were performed, respectively. Firstly, the result from each survey was treated as a separate estimate and a meta-analysis of 18 individual NHIS (*I*^2^ <50% and *P* > 0.1 indicate that there was no significant between-study heterogeneity and a fixed effects model was used, otherwise, a random effects model was used) was conducted to calculate the summary HRs with 95% CIs. Secondly, we conducted a sensitivity analysis by excluding individuals who died within the first 2 years of follow-up. All analyses were conducted using SAS 9.3 (SAS Institute, Cary, NC) and a two-tailed *P* < 0.05 was considered to be statistically significant.

## Results

### Baseline Characteristics

The baseline characteristics of participants across 5 categories of chronic condition status are shown in Table 1. Of the 372,566 participants aged 30–84 years, 27.0, 12.2, 4.4, and 1.1% had 1, 2, 3, and ≥4 chronic diseases, respectively. Those of older age, men, non-Hispanic white, those who had high school education or below, those who were divorced/separated/widowed, those with obesity, former smokers, lifetime abstainer/former drinkers, and those who failed to meet PA guidelines were more likely to experience multimorbidity (all *P* < 0.0001). In addition, the prevalence of multimorbidity was very high in those aged 70–84 years (43.6%), medium among those aged 50–69 years (23.6%), and very low in those aged 30–49 years (5.1%).

**Table 1:** Baseline characteristics according to the number of chronic conditions, NHIS, 1997–2014.

**Characteristic**	**Number of chronic conditions**					***p***
	**0**	**1**	**2**	**3**	**≥4**	
***N*** **(%)**	205,746 (55.2)	100,665 (27.0)	45,478 (12.2)	16,437 (4.4)	4,240 (1.1)	
**Age, years**						
Mean ± SD	46.0 ± 18.5	55.1 ± 20.1	61.8 ± 18.0	65.8 ± 15.9	68.4 ± 12.7	<0.0001
Minimum	30.0	30.0	30.0	30.0	31.0	
Maximum	84.0	84.0	84.0	84.0	84.0	
**BMI, kg/m** ^**2**^						
Mean ± SD	26.5 ± 7.4	28.2 ± 7.3	29.1 ± 7.2	29.6 ± 7.9	29.9 ± 7.0	<0.0001
Minimum	9.9	10.2	10.3	11.7	11.0	
Maximum	45.7	45.7	45.6	45.7	45.6	
**Age category, %**						
30–49	66.7	36.6	18.5	9.2	3.9	<0.0001
50–69	28.4	46.5	50.6	49.0	45.6	
70–84	4.9	16.8	30.9	41.8	50.5	
**Sex, %**						
Men	49.4	47.6	49.7	52.0	52.3	<0.0001
Women	50.6	52.4	50.3	48.0	47.7	
**Race/ethnicity, %**						
non-Hispanic white	70.9	74.9	75.3	76.8	76.7	<0.0001
non-Hispanic black	9.9	12.1	12.6	12.6	13.0	
Hispanic	13.6	8.9	8.6	7.4	7.3	
Others	5.7	4.0	3.5	3.2	3.0	
**Education, %**						
< High school	13.0	15.9	21.4	26.0	31.1	<0.0001
High school	26.9	29.9	31.8	31.3	30.4	
>High school	60.1	54.2	46.8	42.6	38.6	
**Marital status, %**						
Married	67.4	64.6	61.1	59.1	58.2	<0.0001
Divorced/separated/widowed	15.8	22.8	28.7	33.1	34.8	
Never married	16.9	12.6	10.2	7.8	7.0	
**BMI category, %**						
Underweight	1.5	1.2	1.1	1.2	1.1	<0.0001
Normal weight	40.1	28.4	23.5	21.2	19.6	
Overweight	38.0	37.8	36.6	35.0	35.0	
Obese	20.4	32.6	38.8	42.6	44.4	
**Smoking, %**						
Never	57.5	51.4	45.2	40.8	36.9	<0.0001
Former	20.7	28.8	36.5	42.8	48.0	
Current	21.8	19.8	18.3	16.3	15.1	
**Drinking, %**						
Lifetime abstainer	18.4	19.3	22.7	25.0	27.0	<0.0001
Former drinker	12.7	18.1	25.3	34.0	39.8	
Light to moderate	64.0	56.9	47.6	38.0	31.9	
Heavy	4.9	5.7	4.4	3.1	1.4	
**Meeting PA guideline**						
No	52.9	58.9	66.2	73.2	79.1	<0.0001
Yes	47.2	41.1	33.8	26.8	20.9	

*NHIS, national health Interview survey; BMI, body mass index; PA, physical activity*.

### Trends in Prevalence of Chronic Conditions

**Figure 2:**
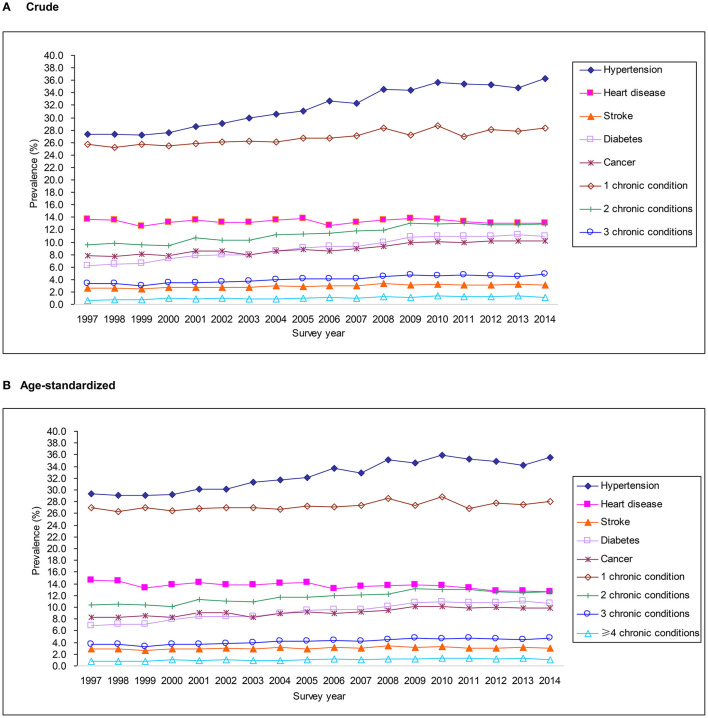
Trends in crude **(A)** and age-standardized **(B)** prevalence of chronic conditions from 1997 to 2014 in the National Health Interview Survey.

The trends in crude and age-standardized prevalence of chronic conditions are shown in Figure 2. The crude prevalence of having 1, 2, 3, and ≥4 chronic conditions increased from 25.8, 9.5, 3.4%, and 0.7% to 28.3, 12.9, 4.9, and 1.2% from 1997 to 2014, while age-standardized prevalence were 27.0, 10.3, 3.7, and 0.7% in 1997, and increased significantly to 28.0, 12.6, 4.7, and 1.1% in 2014, respectively (all *P* for trends <0.0001) (Figures 2A,B). For each specific chronic condition, there was an increased trend in the age-standardized prevalence of hypertension (29.3–35.5%), diabetes (6.9–10.7%), cancer (8.3–9.9%), and stroke (2.8–3.0%) (all *P* for trends <0.0001). However, the trend in heart disease prevalence decreased (*P* for trend <0.0001) (Figure 2B). Similar trends were seen in the crude prevalence of specific chronic condition and multimorbidity (Figure 2A). Subgroup analyses showed that those aged 70–84 years (vs. 50–69 or 30–49 years), men (vs. women), and non-Hispanic black (vs. Hispanics, Non-Hispanic whites or others) had a greater absolute increase in the prevalence of multimorbidity (Supplementary Table 1).

### Survival Analysis of the Association Between Multimorbidity and All-Cause and Cause-Specific Mortality

During a median follow-up of 9.0 years, among 50,309 participants that died from all causes, 11,132 (22.1%) died due to CVD and 13,170 (26.2%) died due to cancer. We found that participants with stroke at baseline had the highest risk of all-cause mortality among all individual chronic conditions (Supplementary Table 2). Compared with participants without any aforementioned chronic conditions, those with 1, 2, 3, and ≥4 of chronic conditions had 1.41 (95% CI 1.37–1.45), 1.94 (1.88–2.00), 2.64 (2.54–2.75), and 3.68 (3.46–3.91) times the risk of all-cause mortality after adjustment for all important covariates (Table 2). Similar patterns were observed for CVD-specific and cancer-specific mortality, with larger effect size for CVD-specific mortality than for cancer-specific mortality (Table 2). Results on association of the number of all covariates with all-cause and cause-specific mortality were presented in Supplementary Table 3. In addition, higher risk of all-cause, CVD-specific, and cancer-specific mortality was also observed across categories of different cardiometabolic multimorbidity after excluding participants who self-reported cancer at baseline (Supplementary Table 4).

**Table 2:** Association of the number of chronic conditions with all-cause and cause-specific mortality.

**Cause of death**	**Number of chronic conditions**				
	**0**	**1**	**2**	**3**	**≥4**
Participants (*N*)	205,746	100,665	45,478	16,437	4,240
**All-cause**					
Deaths (*n*)	14,084	15,834	12,210	6,146	2,035
HRs (95% CIs)	1.00	1.41 (1.37–1.45)	1.94 (1.88–2.00)	2.64 (2.54–2.75)	3.68 (3.46–3.91)
**CVD**					
Deaths (*n*)	2,493	3,347	2,996	1,751	545
HRs (95% CIs)	1.00	1.53 (1.44–1.63)	2.42 (2.26–2.58)	3.91 (3.63–4.22)	5.41 (4.78–6.13)
**Cancer**					
Deaths (*n*)	4,222	4,305	2,942	1,270	431
HRs (95% CIs)	1.00	1.39 (1.31–1.46)	1.86 (1.75–1.97)	2.29 (2.10–2.50)	3.41 (3.00–3.87)

*HR, hazard ratio; CI, confidence interval.*

*Cox proportional hazards regression models were adjusted for sex, age, and race/ethnicity, education, marital status, body mass index, smoking, alcohol intake, and physical activity*.

### Subgroup Analyses

The association between multimorbidity and all-cause mortality stratified by demographic factors and lifestyle factors is shown in Supplementary Table 5. The risk of all-cause mortality was somewhat stronger among those aged 30–49 years or aged 50–69 years (vs. those aged 70–84 years), women (vs. men), those with high school education level (vs. < high school or >high school), those never married (vs. married or divorced/separated/windowed adults), those with obesity (vs. those with normal weight or overweight), and never smokers (vs. former or current smokers).

### Sensitivity Analyses

Sensitivity analysis showed that results from the meta-analysis of all 18 baseline surveys (without significant heterogeneity across the 4 subgroups of different chronic conditions) were similar to our primary results (Supplementary Table 6). In addition, sensitivity analysis that excluded participants who died within the first 2 years of follow-up also showed consistent findings with our primary results (Supplementary Table 7).

## Discussion

Using nationally representative samples of U.S. adults, we found that 17.8% participants had multimorbidity (2 or more chronic conditions). The trends in prevalence of multimorbidity increased significantly from 1997 to 2014. Compared with participants who had no chronic conditions, those with 1, 2, 3, and ≥4 chronic conditions were nearly 1.4, 1.9, 2.6, and 3.7 times more likely to die from all causes. Similar associations were observed for CVD-specific and cancer-specific mortality. Of note, although multimorbidity was more prevalent in participants of older age, the risk of death of those with ≥4 chronic conditions were much higher among both young and middle-aged participants than among older ones. These findings underline the clinical importance of preventing and treating individuals with multimorbidity.

In this study, through 18 survey waves, the prevalence of specific chronic condition increased significantly while the prevalence of heart disease decreased slightly. For heart disease, the leading cause of death in the U.S. (18), although its prevalence has decreased totally, however, the prevalence has increased in older and male participants, which must draw enough attention. Our findings also show an increase in the prevalence of multimorbidity from 1997 to 2014 among U.S. adults. Similar to our findings, several studies have shown the prevalence of multimorbidity among the general population to have become more prevalent (2, 3, 19–21). For example, a study of 65,586 participants aged 50 years or older from the English Longitudinal Study of Aging found that the prevalence of multimorbidity increased from 41.6% in 2002/2003 to 46.6% in 2014/2015 (19). Similar increases in the prevalence of multimorbidity over time were also observed in the U.S., the Netherlands, Canada and Belgium (2, 3, 20, 21). The increase of multimorbidity emphasizes that a complementary strategy of common primary care and personal generalist service is imperative for the medical system (22).

Consistent with our findings, a study conducted using combined data from the Emerging Risk Factors Collaboration (including 689,300 participants with a mean age of 53 years) and from the UK Biobank (including 499,808 participants with a mean age of 57 years) found that having 2 or 3 chronic conditions (including diabetes, stroke, and myocardial infarction) gradually increased the risk of all-cause mortality (e.g., presence of 2: HR 3.6, 95% CI 3.2–4.1; presence of 3: HR 6.0, 95% CI 5.0–7.1) (6). However, hypertension, which is a major factor for mortality (23), was not included in that study. In 2016, a meta-analysis of 26 articles published between 2000 and 2014 including 650,967 older adults aged ≥65 years showed that multimorbidity was associated with increased risk of deaths, with HRs (95% CIs) of 1.73 (95% CI: 1.41–2.13) and 2.72 (95% CI: 1.81–4.08) for people with ≥2 and ≥3 chronic conditions, respectively (4). A recent cohort study of 500,769 participants aged 37–73 years from the UK Biobank also indicated a dose-response relationship between increased number of chronic conditions (including 43 non-cardiometabolic and cardiometabolic conditions) and all-cause, cancer, and vascular mortality (12). However, the participants included in that study were mostly with a lower socioeconomic level than the general UK population.

However, inconsistent with our findings, a study conducted in Hong Kong, China including 4,000 participants aged 65 years or older with a median follow-up of 9 years reported a non-significant association between multimorbidity and mortality (HR 1.20, 95% CI 0.89–1.62) (14). Another study including 1,751 community-dwelling adults in Manitoba aged 65 years or older showed a similar null association (HR 1.00, 95% CI 0.96–1.04) after a median follow-up of 5 years (13). Of note, the sample sizes of these two studies were relatively small, and included participants were all aged 65 years or older. Other possible reasons for the inconsistent findings with ours and others might be due to differences in the sampled population, the chronic diseases studied and confounders that were considered in statistical models.

The current focus in our study for multimorbidity including hypertension, heart disease, stroke, diabetes and cancer is mainly prevalent among older people (24, 25). Similar to previous studies (12, 26), our findings showed that multimorbidity was more likely to occur among those of older age than those of younger age. Among those of old age (≥70 years), the risk of all-cause mortality of those with ≥4 of chronic conditions was 3 fold compared with participants without any above-mentioned chronic conditions. However, among young (30–49 years) and relative older (50–69 years) adults, the risk of all-cause mortality of those with ≥4 of chronic conditions were up to 8 and 6 fold compared with participants without any above-mentioned chronic conditions, suggesting more attention should be paid to young people with multimorbidity. Special interventions for the management of multimorbidity in young adults are warranted.

### Strengths and Limitations

We used the national and representative samples of U.S. adults from 18 baseline surveys with sufficient power (*N* = 372,566) to generate reliable statistical estimates. In addition, we adjusted for several important covariates in the statistical models, and the sensitivity analyses confirmed the stability of our findings. However, our study has several limitations. First, the chronic conditions of participants were self-reported, potentially leading to misclassification of participants with chronic conditions into the group of no chronic conditions, which would likely lead to an underestimation of the strength of the observed association. In addition, the limited information on severity of chronic conditions and the lack of data on accruing multimorbidity exposure change over time might potentially affect our findings. Second, we only focused on the association between the number of chronic conditions and mortality, and did not examine the risk of combinations of specific chronic conditions. However, data from the Emerging Risk Factors Collaboration showed that participants with combinations of any two of three chronic conditions (heart disease, stroke, diabetes) had a similar risk of mortality (6). Third, residual or unmeasured confounding might have influenced our findings. Fourth, although National Death Index has been demonstrated to be accurate in determining overall death previously (15), some other studies have indicated that there was discordance between the NDI cause-specific mortality and other sources of data (27, 28), which might have affected our findings possibly.

## Conclusions

The prevalence of multimorbidity has increased significantly among U.S. adults from 1997 to 2014, especially for older adults, men, and non-Hispanic blacks. The increasing number of chronic conditions was associated with a higher risk of all-cause, CVD-specific, and cancer-specific mortality. In addition, although multimorbidity was more prevalent among those of older age, the association between multimorbidity and mortality was also strong among young and middle-aged adults. As the prevalence of multimorbidity tended to increase over time, our data suggest effective prevention and intervention programs are necessary to limit the increased mortality risk associated with multimorbidity, and that programs should be targeted at both young and older aged adults.

## Data Availability Statement

The original contributions presented in the study are included in the article/Supplementary Material, further inquiries can be directed to the corresponding authors.

## Author Contributions

BX designed the study and is the principal investigator and guarantor. LY conducted the data analysis and drafted the manuscript. CGM, JS, MZ, and BX critically revised the manuscript for important intellectual content. The corresponding author attests that all the listed authors meet the authorship criteria and that no others meeting the criteria have been omitted. All authors approved the final version of the manuscript.

## Funding

BX was supported by the Innovation Team of Climbing Program of Shandong University. CGM was supported by a National Heart Foundation of Australia future leader fellowship (100849). The funders had no role in the study design or implementation; data collection, management, analysis, or interpretation; manuscript preparation, review, or approval; or the decision to submit the manuscript for publication.

## Conflict of Interest

The authors declare that the research was conducted in the absence of any commercial or financial relationships that could be construed as a potential conflict of interest.

## Publisher's Note

All claims expressed in this article are solely those of the authors and do not necessarily represent those of their affiliated organizations, or those of the publisher, the editors and the reviewers. Any product that may be evaluated in this article, or claim that may be made by its manufacturer, is not guaranteed or endorsed by the publisher.
