# Supplementary material for: Trends in Cardiometabolic and Cancer Multimorbidity Prevalence and Its Risk With All-Cause and Cause-Specific Mortality in U.S. Adults: Prospective Cohort Study
Source: Front Cardiovasc Med. 2021 Dec 9;8:731240. doi: 10.3389/fcvm.2021.731240 (PMC8695762; doi:10.3389/fcvm.2021.731240)
Supplement: Supplementary file 1 [file Data_Sheet_1.doc]

# Supplementary Material

| **Supplementary** **Table 1. Trends in prevalence of chronic conditions among U.S. adults by age, sex, and race/ethnicity** | | | | | | | | | | | | | | | | | | | |
| --- | --- | --- | --- | --- | --- | --- | --- | --- | --- | --- | --- | --- | --- | --- | --- | --- | --- | --- | --- |
|  | 1997 | 1998 | 1999 | 2000 | 2001 | 2002 | 2003 | 2004 | 2005 | 2006 | 2007 | 2008 | 2009 | 2010 | 2011 | 2012 | 2013 | 2014 | *P* for trend |
| **30-49 years** |  |  |  |  |  |  |  |  |  |  |  |  |  |  |  |  |  |  |  |
| Hypertension | 15.0 | 14.5 | 14.1 | 14.3 | 15.1 | 16.0 | 15.1 | 15.9 | 16.4 | 17.5 | 17.2 | 18.8 | 18.6 | 19.7 | 19.0 | 18.4 | 18.1 | 18.9 | <.0001 |
| Heart disease | 6.5 | 6.3 | 5.6 | 5.9 | 6.4 | 5.7 | 5.5 | 6.0 | 6.0 | 4.8 | 6.1 | 6.1 | 6.0 | 6.1 | 5.4 | 5.3 | 5.5 | 5.6 | 0.0001 |
| Stroke | 0.5 | 0.5 | 0.7 | 0.7 | 0.7 | 0.7 | 0.8 | 0.7 | 0.7 | 0.8 | 1.0 | 1.2 | 1.1 | 1.1 | 1.0 | 1.0 | 1.0 | 0.9 | <.0001 |
| Diabetes | 2.3 | 2.6 | 2.6 | 3.2 | 3.5 | 3.2 | 3.1 | 3.5 | 4.1 | 4.2 | 3.7 | 4.2 | 5.1 | 4.6 | 4.4 | 4.7 | 4.7 | 4.3 | <.0001 |
| Cancer | 3.1 | 3.3 | 3.0 | 3.2 | 3.6 | 3.1 | 3.0 | 3.2 | 3.7 | 3.5 | 3.6 | 3.6 | 3.5 | 4.1 | 3.2 | 3.7 | 2.9 | 3.7 | <.0001 |
| 1 chronic condition | 18.8 | 18.2 | 17.9 | 18.5 | 19.2 | 19.4 | 18.5 | 18.8 | 19.4 | 20.0 | 19.8 | 21.2 | 20.4 | 21.9 | 19.8 | 20.5 | 19.6 | 21.3 | <.0001 |
| 2 chronic conditions | 3.3 | 3.5 | 3.3 | 3.1 | 3.9 | 3.5 | 3.5 | 4.2 | 4.3 | 4.0 | 4.4 | 4.7 | 5.6 | 5.2 | 5.1 | 5.0 | 4.8 | 4.5 | <.0001 |
| 3 chronic conditions | 0.6 | 0.6 | 0.5 | 0.8 | 0.6 | 0.6 | 0.5 | 0.6 | 0.8 | 0.8 | 0.9 | 0.9 | 0.8 | 0.9 | 0.9 | 0.8 | 0.8 | 0.9 | <.0001 |
| ≥4 chronic conditions | 0.0 | 0.1 | 0.0 | 0.0 | 0.1 | 0.1 | 0.1 | 0.1 | 0.1 | 0.1 | 0.0 | 0.1 | 0.1 | 0.1 | 0.1 | 0.1 | 0.1 | 0.1 | 0.0001 |
| **50-69 years** |  |  |  |  |  |  |  |  |  |  |  |  |  |  |  |  |  |  |  |
| Hypertension | 38.4 | 38.5 | 38.8 | 38.4 | 39.5 | 38.8 | 41.4 | 41.2 | 41.4 | 44.0 | 42.7 | 44.3 | 43.5 | 46.0 | 44.8 | 44.9 | 43.9 | 45.3 | <.0001 |
| Heart disease | 18.0 | 17.8 | 16.4 | 16.5 | 16.8 | 17.1 | 17.0 | 16.9 | 17.6 | 16.4 | 15.8 | 16.0 | 16.7 | 16.2 | 15.8 | 15.6 | 15.6 | 15.0 | <.0001 |
| Stroke | 3.6 | 3.8 | 2.8 | 3.3 | 3.4 | 3.8 | 3.4 | 3.7 | 3.1 | 3.8 | 3.5 | 3.7 | 3.3 | 4.0 | 3.5 | 3.5 | 3.7 | 3.8 | <.0001 |
| Diabetes | 10.2 | 10.3 | 10.1 | 10.9 | 11.5 | 12.0 | 12.1 | 12.5 | 12.9 | 12.8 | 13.3 | 14.1 | 14.3 | 14.5 | 14.8 | 14.6 | 14.7 | 14.5 | <.0001 |
| Cancer | 10.2 | 10.5 | 10.9 | 9.8 | 11.3 | 11.4 | 10.4 | 11.0 | 10.9 | 10.4 | 10.9 | 11.4 | 12.7 | 12.5 | 12.4 | 12.2 | 12.9 | 12.0 | <.0001 |
| 1 chronic condition | 34.0 | 32.9 | 34.3 | 32.9 | 33.4 | 33.3 | 34.1 | 33.6 | 34.0 | 33.1 | 34.0 | 35.1 | 33.4 | 35.6 | 33.4 | 34.3 | 34.3 | 33.9 | <.0001 |
| 2 chronic conditions | 13.6 | 14.3 | 14.0 | 13.8 | 14.8 | 15.4 | 14.4 | 15.5 | 15.5 | 15.9 | 15.9 | 15.7 | 17.2 | 17.0 | 17.2 | 16.7 | 16.6 | 17.0 | <.0001 |
| 3 chronic conditions | 5.0 | 5.1 | 4.5 | 4.5 | 5.0 | 4.9 | 5.5 | 5.3 | 5.3 | 5.8 | 5.1 | 5.7 | 5.7 | 5.8 | 5.9 | 5.8 | 5.7 | 6.0 | <.0001 |
| ≥4 chronic conditions | 1.1 | 1.0 | 0.8 | 1.2 | 1.1 | 1.3 | 1.2 | 1.1 | 1.2 | 1.3 | 1.3 | 1.5 | 1.4 | 1.5 | 1.5 | 1.5 | 1.5 | 1.3 | <.0001 |
| **70-84 years** |  |  |  |  |  |  |  |  |  |  |  |  |  |  |  |  |  |  |  |
| Hypertension | 52.9 | 52.9 | 53.4 | 54.5 | 55.8 | 54.4 | 59.2 | 59.4 | 60.4 | 60.2 | 59.5 | 65.6 | 65.4 | 63.2 | 64.6 | 63.5 | 62.8 | 65.1 | <.0001 |
| Heart disease | 33.4 | 33.3 | 31.3 | 34.1 | 34.3 | 32.9 | 33.6 | 34.6 | 33.7 | 33.7 | 33.4 | 34.2 | 33.5 | 33.6 | 33.8 | 31.4 | 30.7 | 30.8 | 0.0002 |
| Stroke | 8.9 | 8.6 | 8.9 | 9.3 | 9.2 | 8.6 | 9.1 | 10.1 | 10.4 | 9.5 | 9.3 | 10.2 | 9.8 | 8.9 | 9.1 | 9.3 | 9.6 | 7.9 | 0.0004 |
| Diabetes | 13.4 | 13.2 | 14.3 | 15.2 | 16.5 | 16.7 | 16.6 | 18.2 | 18.2 | 19.5 | 19.1 | 18.9 | 20.9 | 23.2 | 21.7 | 20.6 | 22.4 | 21.9 | <.0001 |
| Cancer | 21.5 | 19.1 | 21.7 | 22.0 | 22.0 | 23.7 | 21.3 | 23.4 | 23.5 | 24.1 | 24.3 | 24.4 | 25.8 | 25.1 | 26.4 | 26.2 | 26.0 | 26.0 | <.0001 |
| 1 chronic condition | 35.3 | 35.4 | 36.9 | 35.4 | 34.0 | 34.6 | 36.1 | 34.5 | 35.0 | 34.8 | 34.1 | 35.1 | 33.7 | 32.6 | 32.5 | 34.1 | 35.3 | 34.0 | <.0001 |
| 2 chronic conditions | 25.8 | 24.7 | 24.5 | 24.2 | 27.5 | 24.8 | 26.7 | 27.4 | 27.1 | 28.4 | 28.0 | 28.5 | 28.4 | 28.8 | 28.6 | 27.2 | 27.5 | 28.7 | <.0001 |
| 3 chronic conditions | 11.1 | 10.4 | 10.0 | 11.5 | 11.2 | 12.6 | 12.1 | 13.7 | 13.3 | 12.6 | 13.2 | 14.1 | 15.8 | 14.3 | 15.1 | 14.6 | 13.5 | 14.6 | <.0001 |
| ≥4 chronic conditions | 2.4 | 2.7 | 3.3 | 4.1 | 3.7 | 3.5 | 3.3 | 3.7 | 4.1 | 4.3 | 3.8 | 4.6 | 4.2 | 5.2 | 5.0 | 4.6 | 5.1 | 4.0 | <.0001 |
| **Men** |  |  |  |  |  |  |  |  |  |  |  |  |  |  |  |  |  |  |  |
| Hypertension | 26.3 | 26.4 | 26.7 | 27.1 | 28.4 | 28.6 | 29.3 | 30.6 | 30.3 | 33.5 | 32.3 | 34.3 | 35.2 | 36.7 | 36.5 | 36.7 | 36.1 | 38.0 | <.0001 |
| Heart disease | 13.6 | 13.5 | 13.0 | 13.6 | 14.2 | 13.7 | 13.8 | 14.0 | 14.3 | 13.6 | 14.1 | 13.8 | 15.5 | 14.5 | 14.6 | 14.2 | 14.3 | 14.2 | 0.0003 |
| Stroke | 2.8 | 2.8 | 2.4 | 2.8 | 2.6 | 2.9 | 2.8 | 3.1 | 2.5 | 3.0 | 2.6 | 3.1 | 2.9 | 3.4 | 2.8 | 3.0 | 3.3 | 3.0 | <.0001 |
| Diabetes | 5.9 | 6.5 | 6.4 | 7.7 | 8.3 | 8.6 | 8.6 | 9.2 | 9.4 | 9.6 | 9.3 | 9.8 | 11.5 | 12.0 | 11.5 | 11.4 | 11.9 | 11.4 | <.0001 |
| Cancer | 6.9 | 6.4 | 7.5 | 6.7 | 7.9 | 8.1 | 7.0 | 8.0 | 7.8 | 7.6 | 8.2 | 7.6 | 8.9 | 9.2 | 9.0 | 9.0 | 9.3 | 9.6 | <.0001 |
| 1 chronic condition | 24.6 | 24.3 | 24.4 | 24.3 | 25.0 | 24.8 | 24.8 | 25.2 | 25.2 | 26.4 | 26.2 | 27.5 | 26.3 | 28.8 | 26.3 | 28.1 | 27.8 | 27.3 | <.0001 |
| 2 chronic conditions | 9.3 | 9.1 | 9.4 | 9.7 | 10.8 | 10.3 | 10.4 | 11.1 | 11.5 | 11.6 | 12.1 | 11.6 | 13.5 | 13.0 | 13.5 | 12.9 | 13.2 | 13.7 | <.0001 |
| 3 chronic conditions | 3.2 | 3.4 | 3.4 | 3.4 | 3.6 | 4.1 | 3.9 | 4.4 | 4.0 | 4.3 | 4.1 | 4.2 | 5.3 | 4.9 | 5.2 | 5.0 | 5.0 | 5.5 | <.0001 |
| ≥4 chronic conditions | 0.7 | 0.7 | 0.7 | 1.0 | 1.0 | 1.0 | 1.0 | 1.1 | 1.0 | 1.2 | 0.9 | 1.2 | 1.2 | 1.5 | 1.3 | 1.3 | 1.4 | 1.2 | <.0001 |
| **Women** |  |  |  |  |  |  |  |  |  |  |  |  |  |  |  |  |  |  |  |
| Hypertension | 28.4 | 28.1 | 27.7 | 27.9 | 28.7 | 29.4 | 30.5 | 30.4 | 31.8 | 31.8 | 32.2 | 34.9 | 33.6 | 34.7 | 34.4 | 34.0 | 33.6 | 34.6 | <.0001 |
| Heart disease | 13.8 | 13.6 | 12.0 | 12.8 | 12.9 | 12.6 | 12.5 | 13.1 | 13.2 | 11.8 | 12.4 | 13.2 | 12.0 | 12.7 | 12.1 | 11.8 | 11.8 | 11.8 | <.0001 |
| Stroke | 2.5 | 2.5 | 2.5 | 2.6 | 2.9 | 2.6 | 2.7 | 2.9 | 3.1 | 3.0 | 3.3 | 3.5 | 3.3 | 3.1 | 3.3 | 3.3 | 3.2 | 3.1 | <.0001 |
| Diabetes | 6.6 | 6.4 | 6.6 | 6.9 | 7.4 | 7.3 | 7.3 | 8.0 | 8.7 | 9.1 | 9.2 | 10.0 | 10.0 | 9.8 | 10.3 | 10.5 | 10.6 | 10.5 | <.0001 |
| Cancer | 8.6 | 9.0 | 8.6 | 9.0 | 9.2 | 9.2 | 8.8 | 9.1 | 9.9 | 9.5 | 9.8 | 10.9 | 11.0 | 11.0 | 10.9 | 11.4 | 11.1 | 10.9 | <.0001 |
| 1 chronic condition | 26.9 | 26.1 | 26.9 | 26.6 | 26.6 | 27.3 | 27.5 | 26.9 | 28.2 | 26.9 | 27.9 | 29.1 | 28.1 | 28.6 | 27.6 | 27.9 | 27.9 | 29.3 | <.0001 |
| 2 chronic conditions | 9.7 | 10.5 | 9.7 | 9.2 | 10.6 | 10.4 | 10.1 | 11.3 | 11.0 | 11.3 | 11.5 | 12.3 | 12.7 | 12.7 | 12.7 | 12.6 | 12.3 | 12.2 | <.0001 |
| 3 chronic conditions | 3.6 | 3.3 | 2.7 | 3.5 | 3.3 | 3.1 | 3.7 | 3.6 | 4.1 | 3.9 | 4.0 | 4.6 | 4.1 | 4.2 | 4.3 | 4.3 | 4.1 | 4.3 | <.0001 |
| ≥4 chronic conditions | 0.7 | 0.7 | 0.7 | 0.9 | 0.8 | 0.9 | 0.7 | 0.8 | 1.0 | 1.0 | 1.0 | 1.2 | 1.0 | 1.1 | 1.2 | 1.2 | 1.3 | 1.1 | <.0001 |
| **Non-Hispanic white** |  |  |  |  |  |  |  |  |  |  |  |  |  |  |  |  |  |  |  |
| Hypertension | 27.2 | 26.9 | 26.9 | 27.3 | 28.1 | 28.9 | 29.9 | 30.9 | 31.2 | 33.2 | 32.5 | 35.1 | 34.8 | 35.7 | 35.9 | 35.7 | 35.5 | 36.9 | <.0001 |
| Heart disease | 14.9 | 14.7 | 13.4 | 14.2 | 14.7 | 14.3 | 14.6 | 15.1 | 15.5 | 14.3 | 14.8 | 15.4 | 15.7 | 15.3 | 14.8 | 14.7 | 15.0 | 14.9 | <.0001 |
| Stroke | 2.7 | 2.7 | 2.5 | 2.7 | 2.7 | 2.8 | 2.8 | 3.1 | 2.9 | 3.0 | 2.9 | 3.4 | 3.3 | 3.3 | 2.9 | 3.1 | 3.4 | 3.1 | <.0001 |
| Diabetes | 5.4 | 5.6 | 5.8 | 6.5 | 7.1 | 7.2 | 7.4 | 7.8 | 8.5 | 8.5 | 8.0 | 9.1 | 9.9 | 9.8 | 10.1 | 9.7 | 10.4 | 9.8 | <.0001 |
| Cancer | 9.0 | 9.0 | 9.4 | 9.3 | 10.3 | 10.4 | 9.4 | 10.2 | 10.7 | 10.4 | 11.0 | 11.3 | 12.5 | 12.3 | 12.3 | 12.6 | 12.7 | 12.9 | <.0001 |
| 1 chronic condition | 26.6 | 25.5 | 26.3 | 26.0 | 26.5 | 26.6 | 26.8 | 26.8 | 27.5 | 27.7 | 28.0 | 29.6 | 27.9 | 29.5 | 27.7 | 29.4 | 29.2 | 29.5 | <.0001 |
| 2 chronic conditions | 9.7 | 10.1 | 9.7 | 9.7 | 10.8 | 10.5 | 10.8 | 11.7 | 11.8 | 11.8 | 12.2 | 12.4 | 14.1 | 13.3 | 13.7 | 13.1 | 13.7 | 13.5 | <.0001 |
| 3 chronic conditions | 3.5 | 3.5 | 3.1 | 3.5 | 3.5 | 3.9 | 4.1 | 4.4 | 4.3 | 4.5 | 4.2 | 4.7 | 5.0 | 4.9 | 5.2 | 4.9 | 4.8 | 5.4 | <.0001 |
| ≥4 chronic conditions | 0.7 | 0.7 | 0.8 | 1.0 | 1.0 | 1.0 | 0.9 | 0.9 | 1.2 | 1.1 | 1.0 | 1.4 | 1.3 | 1.4 | 1.3 | 1.3 | 1.4 | 1.2 | <.0001 |
| **Non-Hispanic black** |  |  |  |  |  |  |  |  |  |  |  |  |  |  |  |  |  |  |  |
| Hypertension | 37.1 | 36.6 | 36.5 | 36.7 | 38.1 | 38.6 | 41.5 | 38.6 | 40.1 | 42.3 | 42.2 | 43.5 | 43.4 | 48.1 | 45.5 | 47.0 | 44.6 | 48.0 | <.0001 |
| Heart disease | 11.5 | 11.2 | 11.1 | 11.5 | 12.4 | 11.1 | 10.9 | 10.6 | 11.9 | 11.0 | 11.0 | 11.0 | 11.8 | 11.7 | 12.2 | 11.8 | 11.3 | 11.7 | 0.0054 |
| Stroke | 3.4 | 3.2 | 3.8 | 3.2 | 3.8 | 3.4 | 4.0 | 3.5 | 3.8 | 5.1 | 4.1 | 4.0 | 4.3 | 4.5 | 4.9 | 4.7 | 3.9 | 4.8 | <.0001 |
| Diabetes | 10.8 | 10.2 | 10.0 | 11.5 | 11.9 | 11.6 | 11.6 | 12.1 | 12.7 | 13.5 | 13.7 | 13.2 | 15.3 | 14.7 | 14.4 | 16.4 | 14.9 | 16.5 | <.0001 |
| Cancer | 4.2 | 4.1 | 4.1 | 3.6 | 3.8 | 3.5 | 4.3 | 4.5 | 4.1 | 4.5 | 5.0 | 4.4 | 4.7 | 5.9 | 5.6 | 5.7 | 6.2 | 5.5 | <.0001 |
| 1 chronic condition | 27.5 | 29.9 | 26.5 | 28.7 | 27.7 | 29.1 | 30.3 | 29.3 | 30.2 | 29.8 | 28.6 | 30.7 | 29.8 | 33.9 | 30.3 | 30.7 | 29.9 | 31.5 | <.0001 |
| 2 chronic conditions | 11.3 | 10.2 | 11.9 | 10.8 | 13.2 | 12.6 | 11.8 | 13.0 | 12.2 | 12.9 | 13.8 | 13.3 | 13.5 | 14.4 | 15.3 | 15.7 | 14.2 | 15.6 | <.0001 |
| 3 chronic conditions | 4.4 | 3.7 | 3.9 | 3.9 | 4.2 | 3.5 | 4.5 | 3.4 | 4.6 | 5.0 | 5.0 | 4.9 | 5.9 | 5.3 | 5.0 | 5.8 | 5.2 | 5.2 | <.0001 |
| ≥4 chronic conditions | 0.9 | 1.0 | 0.8 | 1.1 | 0.8 | 0.8 | 1.2 | 1.0 | 1.0 | 1.4 | 1.2 | 0.9 | 1.2 | 1.6 | 1.7 | 1.5 | 1.7 | 2.0 | <.0001 |
| **Hispanic** |  |  |  |  |  |  |  |  |  |  |  |  |  |  |  |  |  |  |  |
| Hypertension | 20.7 | 21.1 | 21.6 | 20.0 | 23.6 | 23.2 | 21.5 | 22.4 | 23.4 | 23.4 | 23.7 | 25.4 | 26.7 | 28.2 | 27.7 | 26.7 | 26.3 | 28.8 | <.0001 |
| Heart disease | 8.2 | 8.4 | 8.3 | 7.9 | 7.4 | 7.5 | 7.1 | 7.8 | 7.0 | 6.9 | 8.5 | 7.1 | 7.9 | 7.9 | 8.3 | 7.4 | 7.8 | 7.1 | <.0001 |
| Stroke | 1.9 | 2.0 | 1.1 | 1.8 | 2.2 | 2.3 | 1.6 | 2.3 | 1.6 | 1.6 | 2.3 | 2.5 | 1.9 | 2.4 | 2.5 | 2.3 | 2.3 | 2.2 | 0.0001 |
| Diabetes | 8.2 | 9.3 | 8.3 | 9.0 | 9.7 | 9.9 | 8.4 | 9.3 | 9.6 | 10.7 | 11.0 | 10.7 | 12.7 | 13.5 | 12.7 | 12.6 | 12.6 | 12.9 | <.0001 |
| Cancer | 3.0 | 3.2 | 2.8 | 3.0 | 3.1 | 2.8 | 2.8 | 3.1 | 3.2 | 3.3 | 3.4 | 3.4 | 3.7 | 4.1 | 3.5 | 3.7 | 3.9 | 4.1 | 0.0001 |
| 1 chronic condition | 18.7 | 19.2 | 21.1 | 19.1 | 21.3 | 21.6 | 20.8 | 19.4 | 19.6 | 20.6 | 20.5 | 20.0 | 21.6 | 22.4 | 21.6 | 20.8 | 21.8 | 23.3 | <.0001 |
| 2 chronic conditions | 7.4 | 7.4 | 6.8 | 7.0 | 7.9 | 7.6 | 7.1 | 7.5 | 8.1 | 8.3 | 9.0 | 8.8 | 9.7 | 11.1 | 9.5 | 9.5 | 8.9 | 9.6 | <.0001 |
| 3 chronic conditions | 2.0 | 2.7 | 2.1 | 2.1 | 2.4 | 2.1 | 1.4 | 2.5 | 2.6 | 2.2 | 2.5 | 2.8 | 3.2 | 2.6 | 3.3 | 3.3 | 3.0 | 3.3 | <.0001 |
| ≥4 chronic conditions | 0.6 | 0.4 | 0.3 | 0.5 | 0.5 | 0.6 | 0.5 | 0.8 | 0.3 | 0.5 | 0.7 | 0.7 | 0.5 | 0.9 | 1.0 | 0.7 | 1.1 | 0.6 | <.0001 |
| **Others** |  |  |  |  |  |  |  |  |  |  |  |  |  |  |  |  |  |  |  |
| Hypertension | 19.9 | 23.6 | 20.9 | 25.0 | 23.6 | 19.1 | 19.9 | 24.3 | 27.1 | 25.3 | 27.4 | 30.9 | 27.4 | 28.5 | 27.8 | 28.5 | 28.8 | 26.2 | 0.0001 |
| Heart disease | 8.3 | 9.2 | 8.7 | 8.9 | 8.4 | 9.3 | 7.0 | 8.5 | 6.7 | 7.2 | 8.1 | 8.3 | 6.5 | 9.0 | 8.4 | 8.3 | 6.8 | 7.9 | <.0001 |
| Stroke | 1.5 | 2.2 | 1.6 | 2.6 | 2.5 | 1.9 | 1.7 | 2.0 | 1.5 | 1.5 | 2.8 | 1.9 | 0.8 | 2.6 | 3.3 | 2.6 | 2.2 | 1.8 | 0.0096 |
| Diabetes | 6.5 | 6.2 | 7.3 | 7.4 | 7.6 | 8.7 | 7.7 | 10.5 | 7.5 | 8.9 | 13.1 | 11.1 | 8.3 | 11.3 | 10.8 | 10.6 | 11.0 | 9.9 | 0.0001 |
| Cancer | 3.6 | 2.6 | 3.4 | 3.9 | 1.6 | 2.6 | 3.8 | 3.3 | 3.3 | 5.3 | 3.6 | 5.7 | 3.8 | 4.2 | 4.3 | 4.6 | 3.2 | 3.6 | 0.0109 |
| 1 chronic condition | 20.3 | 18.6 | 21.2 | 23.0 | 18.7 | 18.6 | 18.7 | 21.6 | 22.6 | 19.9 | 25.5 | 25.3 | 25.4 | 22.9 | 23.5 | 23.1 | 22.5 | 21.0 | 0.0055 |
| 2 chronic conditions | 4.8 | 8.2 | 6.4 | 6.4 | 7.2 | 7.1 | 5.6 | 8.2 | 7.6 | 10.2 | 7.8 | 10.0 | 7.6 | 9.4 | 9.3 | 9.7 | 8.6 | 9.5 | 0.0002 |
| 3 chronic conditions | 2.7 | 1.2 | 2.4 | 2.9 | 3.2 | 2.5 | 2.1 | 2.4 | 2.4 | 2.2 | 3.7 | 2.9 | 1.6 | 3.7 | 2.6 | 2.7 | 3.5 | 2.6 | 0.0083 |
| ≥4 chronic conditions | 0.5 | 1.3 | 0.1 | 0.8 | 0.3 | 0.3 | 0.9 | 0.8 | 0.3 | 0.3 | 0.7 | 1.0 | 0.3 | 0.7 | 1.2 | 1.0 | 0.4 | 0.4 | 0.0110 |

Multivariable survey logistic regression analysis was used to examine the trends in prevalence of chronic conditions with adjustment for sex, age, race/ethnicity, education, marital status, body mass index, smoking, alcohol intake, and physical activity

*P*<0.0006 (0.05/81) was considered statistically significant.

| **Supplementary** **Table 2. Association of individual chronic conditions with all-cause and cause-specific mortality** | | | | | |
| --- | --- | --- | --- | --- | --- |
| Outcome | Hypertension | Heart disease | Stroke | Diabetes | Cancer |
| All-cause mortality | 1.36 (1.33-1.39) | 1.58 (1.55-1.62) | 1.83 (1.77-1.89) | 1.72 (1.67-1.77) | 1.45 (1.41-1.50) |
| CVD mortality | 1.64 (1.57-1.72) | 2.27 (2.16-2.37) | 2.62 (2.44-2.80) | 1.92 (1.81-2.04) | 1.06 (0.99-1.13) |
| Cancer mortality | 1.15 (1.10-1.20) | 1.24 (1.19-1.30) | 1.40 (1.29-1.52) | 1.36 (1.28-1.45) | 2.62 (2.50-2.76) |
| Data are expressed as hazard ratio (95% confidence interval) | | | | | |
| Cox proportional hazards regression models were adjusted for sex, age, race/ethnicity, education, marital status, body mass index, smoking, alcohol intake, and physical activity. | | | | | |

| **Supplementary** **Table 3. Association of the number of chronic conditions and all covariates with all-cause and cause-specific mortality** | | | | | | | | |
| --- | --- | --- | --- | --- | --- | --- | --- | --- |
| Variables | All-cause | |  | CVD | |  | Cancer | |
|  | HR | 95% CI |  | HR | 95% CI |  | HR | 95% CI |
| **Number of chronic conditions** |  |  |  |  |  |  |  |  |
| 0 | 1.00 |  |  | 1.00 |  |  | 1.00 |  |
| 1 | 1.41 | 1.37-1.45 |  | 1.53 | 1.44-1.63 |  | 1.39 | 1.31-1.46 |
| 2 | 1.94 | 1.88-2.00 |  | 2.42 | 2.26-2.58 |  | 1.86 | 1.75-1.97 |
| 3 | 2.64 | 2.54-2.75 |  | 3.91 | 3.63-4.22 |  | 2.29 | 2.10-1.50 |
| ≥4 | 3.68 | 3.46-3.91 |  | 5.41 | 4.78-6.13 |  | 3.41 | 3.00-3.87 |
| **Sex** |  |  |  |  |  |  |  |  |
| Male | 1.00 |  |  | 1.00 |  |  | 1.00 |  |
| Female | 0.67 | 0.65-0.68 |  | 0.53 | 0.51-0.56 |  | 0.68 | 0.65-0.71 |
| **Age** | 1.08 | 1.08-1.08 |  | 1.10 | 1.10-1.11 |  | 1.08 | 1.08-1.08 |
| **Race** |  |  |  |  |  |  |  |  |
| Hispanic | 1.00 |  |  | 1.00 |  |  | 1.00 |  |
| Non-Hispanic white | 1.04 | 0.99-1.09 |  | 1.11 | 1.02-1.21 |  | 1.25 | 1.15-1.36 |
| Non-Hispanic black | 1.11 | 1.05-1.16 |  | 1.20 | 1.10-1.32 |  | 1.28 | 1.16-1.41 |
| Others | 0.98 | 0.90-1.07 |  | 0.94 | 0.80-1.11 |  | 1.25 | 1.08-1.45 |
| **Marital status** |  |  |  |  |  |  |  |  |
| Married | 1.00 |  |  | 1.00 |  |  | 1.00 |  |
| Divorced/separated/widowed | 1.25 | 1.22-1.28 |  | 1.31 | 1.25-1.37 |  | 1.09 | 1.04-1.14 |
| Never married | 1.39 | 1.33-1.44 |  | 1.55 | 1.43-1.69 |  | 1.02 | 0.95-1.10 |
| **Education** |  |  |  |  |  |  |  |  |
| <High school | 1.00 |  |  | 1.00 |  |  | 1.00 |  |
| High school | 0.87 | 0.85-0.90 |  | 0.80 | 0.75-0.85 |  | 0.85 | 0.81-0.90 |
| >High school | 0.76 | 0.74-0.78 |  | 0.68 | 0.64-0.72 |  | 0.73 | 0.70-0.77 |
| **BMI** | 0.98 | 0.98-0.99 |  | 0.99 | 0.98-0.99 |  | 0.98 | 0.97-0.98 |
| **Smoking status** |  |  |  |  |  |  |  |  |
| Never | 1.00 |  |  | 1.00 |  |  | 1.00 |  |
| Former | 1.32 | 1.29-1.36 |  | 1.22 | 1.16-1.29 |  | 1.57 | 1.49-1.65 |
| Current | 2.19 | 2.13-2.26 |  | 2.41 | 2.26-2.58 |  | 2.91 | 2.74-3.08 |
| **Drinking** |  |  |  |  |  |  |  |  |
| Lifetime abstainer | 1.00 |  |  | 1.00 |  |  | 1.00 |  |
| Former drinker | 1.07 | 1.04-1.10 |  | 1.06 | 0.99-1.13 |  | 1.10 | 1.03-1.17 |
| Light to moderate | 0.79 | 0.77-0.81 |  | 0.74 | 0.69-0.79 |  | 0.82 | 0.78-0.87 |
| Heavy | 1.04 | 0.99-1.10 |  | 0.84 | 0.75-0.95 |  | 1.16 | 1.06-1.27 |
| **Meeting PA guideline** |  |  |  |  |  |  |  |  |
| No | 1.00 |  |  | 1.00 |  |  | 1.00 |  |
| Yes | 0.69 | 0.67-0.71 |  | 0.63 | 0.59-0.66 |  | 0.74 | 0.71-0.77 |
| NHIS, National Health Interview Survey; BMI, body mass index; PA, physical activity | | | | | | | | |
| Data are expressed as hazard ratio (95% confidence interval) | | | | | | | | |
| Cox proportional hazards regression models were adjusted for sex, age, race/ethnicity, education, marital status, body mass index, smoking, alcohol intake, and physical activity when appropriate. | | | | | | | | |

| **Supplementary** **Table 4. Association of the number of cardiometabolic chronic conditions with all-cause and cause-specific mortality after excluding participants with self-reported cancer at baseline** | | | | | |
| --- | --- | --- | --- | --- | --- |
| Cause of death | Number of cardiometabolic chronic conditions | | | | |
|  | 0 | 1 | 2 | 3 | 4 |
| Participants (N) | 218567 | 99485 | 40586 | 12020 | 1908 |
| **All-cause** |  |  |  |  |  |
| Deaths (n) | 16509 | 16712 | 11356 | 4477 | 988 |
| HRs (95% CIs) | 1.00 | 1.33 (1.29-1.37) | 1.84 (1.78-1.90) | 2.68 (2.57-2.79) | 4.04 (3.71-4.41) |
| **CVD** |  |  |  |  |  |
| Deaths (n) | 2788 | 3619 | 2984 | 1425 | 316 |
| HRs (95% CIs) | 1.00 | 1.59 (1.50-1.69) | 2.64 (2.48-2.82) | 4.72 (4.34-5.13) | 7. 80 (6.66-9.14) |
| **Cancer** |  |  |  |  |  |
| Deaths (n) | 5436 | 4426 | 2379 | 792 | 137 |
| HRs (95% CIs) | 1.00 | 1.13 (1.07-1.18) | 1.35 (1.28-1.44) | 1.64 (1.50-1.80) | 2.15 (1.75-2.65) |
| HR, hazard ratio; CI, confidence interval | | | | | |
| Cox proportional hazards regression models were adjusted for sex, age, and race/ethnicity, education, marital status, body mass index, smoking, alcohol intake, and physical activity | | | | | |

| **Supplementary** **Table 5. Subgroup analyses of the association of the number of chronic conditions with all-cause mortality** | | | | | | |
| --- | --- | --- | --- | --- | --- | --- |
| Subgroup | Number of chronic conditions | | | | | *P* for interaction |
|  | 0 | 1 | 2 | 3 | ≥4 |  |
| **Age, yrs,** |  |  |  |  |  | <0.001 |
| 30-49 | 1.00 | 1.86 (1.74-1.99) | 3.18 (2.87-3.53) | 5.11 (4.30-6.06) | 8.78 (5.95-12.95) |  |
| 50-69 | 1.00 | 1.57 (1.50-1.64) | 2.60 (2.47-2.73) | 4.06 (3.81-4.33) | 6.10 (5.51-6.77) |  |
| 70-84 | 1.00 | 1.30 (1.25-1.36) | 1.68 (1.61-1.76) | 2.22 (2.09-2.34) | 3.15 (2.92-3.40) |  |
| **Sex** |  |  |  |  |  | <0.001 |
| Men | 1.00 | 1.40 (1.35-1.46) | 1.94 (1.86-2.03) | 2.53 (2.40-2.68) | 3.38 (3.10-3.68) |  |
| Women | 1.00 | 1.42 (1.36-1.48) | 1.94 (1.85-2.04) | 2.79 (2.63-2.96) | 4.12 (3.79-4.47) |  |
| **Education** |  |  |  |  |  | <0.001 |
| <High school | 1.00 | 1.32 (1.25-1.39) | 1.71 (1.62-1.81) | 2.33 (2.17-2.51) | 3.13 (2.84-3.46) |  |
| High school | 1.00 | 1.40 (1.34-1.47) | 2.02 (1.91-2.13) | 2.83 (2.63-3.04) | 4.29 (3.82-4.82) |  |
| >High school | 1.00 | 1.45 (1.38-1.51) | 2.02 (1.92-2.12) | 2.67 (2.49-2.86) | 3.61 (3.25-4.03) |  |
| **Marital status** |  |  |  |  |  | 0.003 |
| Married | 1.00 | 1.40 (1.34-1.45) | 1.97 (1.89-2.06) | 2.74 (2.58-2.90) | 3.73 (3.42-4.06) |  |
| Divorced/separated/widowed | 1.00 | 1.36 (1.31-1.42) | 1.81 (1.73-1.90) | 2.37 (2.24-2.50) | 3.49 (3.21-3.79) |  |
| Never married | 1.00 | 1.56 (1.43-1.69) | 2.14 (1.94-2.37) | 3.16 (2.75-3.62) | 4.04 (3.07-5.32) |  |
| **BMI, kg/m^2^** |  |  |  |  |  | 0.007 |
| Underweight | 1.00 | 1.41 (1.23-1.63) | 1.66 (1.39-1.98) | 2.39 (1.84-3.11) | 2.76 (1.91-3.99) |  |
| Normal weight | 1.00 | 1.41 (1.34-1.47) | 1.88 (1.78-1.99) | 2.47 (2.30-2.66) | 3.62 (3.22-4.08) |  |
| Overweight | 1.00 | 1.36 (1.30-1.43) | 1.90 (1.81-2.00) | 2.52 (2.36-2.70) | 3.42 (3.09-3.79) |  |
| Obese | 1.00 | 1.43 (1.34-1.52) | 2.05 (1.92-2.19) | 2.98 (2.76-3.22) | 4.25 (3.83-4.72) |  |
| **Smoking** |  |  |  |  |  | 0.002 |
| Never | 1.00 | 1.34 (1.28-1.40) | 1.92 (1.83-2.01) | 2.66 (2.50-2.83) | 4.14 (3.76-4.55) |  |
| Former | 1.00 | 1.38 (1.31-1.45) | 1.89 (1.80-1.99) | 2.59 (2.43-2.76) | 3.33 (3.02-3.67) |  |
| Current | 1.00 | 1.54 (1.46-1.62) | 2.01 (1.89-2.14) | 2.60 (2.38-2.83) | 3.61 (3.13-4.17) |  |
| NHIS, National Health Interview Survey; BMI, body mass index | | | | | | |
| Data are expressed as hazard ratio (95% confidence interval) | | | | | | |
| Cox proportional hazards regression models were adjusted for sex, age, race/ethnicity, education, marital status, body mass index, smoking, alcohol intake, and physical activity when appropriate. | | | | | | |

| **Supplementary** **Table 6. Meta-analyses of the association of the number of chronic conditions with all-cause mortality** | | | | | | |
| --- | --- | --- | --- | --- | --- | --- |
| Survey year | N | Number of chronic conditions | | | | |
|  |  | 0 | 1 | 2 | 3 | ≥4 |
| 1997 | 24,868 | 1.00 | 1.39 (1.30-1.48) | 1.89 (1.75-2.04) | 2.35 (2.09-2.64) | 3.41 (2.77-4.21) |
| 1998 | 21,896 | 1.00 | 1.34 (1.25-1.45) | 1.92 (1.76-2.10) | 2.60 (2.33-2.90) | 4.05 (3.33-4.93) |
| 1999 | 20,561 | 1.00 | 1.42 (1.31-1.54) | 1.91 (1.74-2.10) | 2.63 (2.29-3.02) | 3.16 (2.50-3.99) |
| 2000 | 21,288 | 1.00 | 1.40 (1.28-1.52) | 1.91 (1.73-2.11) | 2.41 (2.11-2.75) | 3.34 (2.59-4.31) |
| 2001 | 21,758 | 1.00 | 1.42 (1.30-1.55) | 2.10 (1.90-2.31) | 2.94 (2.57-3.36) | 3.64 (2.90-4.58) |
| 2002 | 20,107 | 1.00 | 1.35 (1.21-1.50) | 1.98 (1.77-2.21) | 2.62 (2.24-3.06) | 4.45 (3.60-5.51) |
| 2003 | 19,732 | 1.00 | 1.32 (1.19-1.48) | 1.88 (1.67-2.12) | 2.79 (2.36-3.30) | 3.85 (2.97-4.99) |
| 2004 | 20,681 | 1.00 | 1.54 (1.36-1.73) | 2.12 (1.87-2.42) | 2.79 (2.39-3.26) | 4.46 (3.55-5.61) |
| 2005 | 20,486 | 1.00 | 1.41 (1.24-1.60) | 1.90 (1.67-2.17) | 2.93 (2.49-3.45) | 4.09 (3.26-5.14) |
| 2006 | 15,937 | 1.00 | 1.51 (1.30-1.75) | 2.12 (1.80-2.49) | 3.14 (2.56-3.85) | 3.86 (2.98-5.00) |
| 2007 | 15,466 | 1.00 | 1.47 (1.24-1.74) | 1.84 (1.53-2.21) | 2.58 (2.04-3.27) | 3.74 (2.87-4.89) |
| 2008 | 14,840 | 1.00 | 1.32 (1.10-1.60) | 2.12 (1.74-2.57) | 2.97 (2.36-3.75) | 3.97 (2.90-5.43) |
| 2009 | 19,231 | 1.00 | 1.45 (1.19-1.75) | 1.88 (1.55-2.28) | 2.45 (1.92-3.14) | 3.33 (2.37-4.68) |
| 2010 | 18,863 | 1.00 | 1.63 (1.33-1.98) | 1.98 (1.56-2.52) | 2.88 (2.28-3.64) | 4.33 (3.16-5.95) |
| 2011 | 22,951 | 1.00 | 1.26 (1.02-1.55) | 1.66 (1.32-2.09) | 2.37 (1.80-3.13) | 3.82 (2.85-5.13) |
| 2012 | 24,065 | 1.00 | 1.49 (1.16-1.90) | 2.07 (1.62-2.66) | 3.15 (2.31-4.29) | 3.32 (2.26-4.90) |
| 2013 | 24,132 | 1.00 | 1.24 (0.92-1.67) | 1.75 (1.25-2.45) | 2.88 (1.99-4.16) | 2.78 (1.74-4.43) |
| 2014 | 25,704 | 1.00 | 2.22 (1.42-3.48) | 3.15 (2.03-4.88) | 3.48 (2.06-5.86) | 5.98 (3.11-11.50) |
| *I*^2^ (%) |  |  | 0.0 | 0.0 | 6.4 | 0.0 |
| *P for heterogeneity* |  |  | 0.555 | 0.604 | 0.378 | 0.616 |
| Statistical model |  |  | Fixed | Fixed | Fixed | Fixed |
| Meta-analysis | 372,566 | 1.00 | 1.40 (1.36-1.44) | 1.95 (1.89-2.01) | 2.67 (2.57-2.78) | 3.81 (3.58-4.05) |
| Data are expressed as hazard ratio (95% confidence interval) | | | | | | |
| Cox proportional hazards regression models were adjusted for sex, age, race/ethnicity, education, marital status, body mass index, smoking, alcohol intake, and physical activity. | | | | | | |

| **Supplementary** **Table 7. Association of the number of chronic conditions with all-cause and cause-specific mortality after excluding participants who died within the first 2 years of follow-up** | | | | | |
| --- | --- | --- | --- | --- | --- |
| Cause of death | Number of chronic conditions | | | | |
|  | 0 | 1 | 2 | 3 | ≥4 |
| All-cause | 1.00 | 1.36 (1.33-1.40) | 1.88 (1.82-1.94) | 2.48 (2.38-2.59) | 3.47 (3.24-3.72) |
| CVD | 1.00 | 1.55 (1.45-1.65) | 2.41 (2.25-2.59) | 3.85 (3.54-4.18) | 5.26 (4.58-6.03) |
| Cancer | 1.00 | 1.29 (1.22-1.36) | 1.70 (1.60-1.81) | 1.90 (1.73-2.09) | 2.99 (2.59-3.46) |
| Data are expressed as hazard ratio (95% confidence interval) | | | | | |
| Cox proportional hazards regression models were adjusted for sex, age, race/ethnicity, education, marital status, body mass index, smoking, alcohol intake, and physical activity. | | | | | |
